# Measuring the Impact of Public Health Policys

**Published:** 2010-06-15

**Authors:** Ross C. Brownson, Rachel Seiler, Amy A. Eyler

**Affiliations:** Prevention Research Center in St Louis, George Warren Brown School of Social Work, Washington University in St Louis. Dr Brownson is also affiliated with the Department of Surgery and Alvin J. Siteman Cancer Center, Washington University School of Medicine, Washington University in St Louis, Saint Louis, Missouri.; Prevention Research Center in St Louis, Saint Louis University School of Public Health, Saint Louis, Missouri; Department of Surgery and Alvin J. Siteman Cancer Center, Washington University School of Medicine, Washington University in St Louis, Saint Louis, Missouri

## Abstract

Effective health policies and allocation of public health resources can substantially improve public health. An objective of public health practitioners and researchers is to identify key metrics that would help improve effective policies and terminate poor ones. We review articles published in 2008 surrounding measurement issues for public health policy and present a set of recommendations for future emphasis. We found that a set of consensus metrics for population health performance should be developed. However, considerable work is needed to develop appropriate metrics covering policy approaches that can affect large populations, intervention approaches within organizations, and individual-level behavioral approaches for prevention or disease management.

## Introduction

Effective health policies and allocation of public health resources can substantially improve public health (1). For example, each of the 10 great public health achievements of the 20th century (2) was influenced by policy change, such as seat belt laws or regulations governing permissible workplace exposures. To improve public health outcomes, evidence-based policy is developed through a continuous process that uses the best available quantitative and qualitative evidence (3). To broaden the evidence base, a "pay-for-performance" concept that has been widely applied to medical care (4) should be considered for population- and policy-related outcomes (5). In the pay-for-performance approach, providers are rewarded for meeting targets for health care services. For public health, the analogous example might be if public health laws were based in part on policies that are the most cost-effective.

A difference between individual-level health care and population-level approaches for improving health is that public health interventions often occur at multiple levels (6). Upstream interventions involve policy approaches that can affect large populations through regulation, increased access, or economic incentives. For example, increasing tobacco taxes is an effective method for controlling tobacco-related diseases (7). Midstream interventions occur within organizations. For example, worksite-based programs that increase employee access to facilities for physical activity show promise in improving health. Most research has been conducted on downstream interventions, which often involve individual-level behavioral approaches for prevention or disease management. A set of metrics (ie, a group of related measures to quantify some characteristic) can be developed corresponding to these 3 levels. For example, for tobacco control, 3 metrics might be the number of state laws that ban smoking (upstream), the number of private worksites that ban smoking in states with weak laws (midstream), and the rate of self-reported exposure to secondhand smoke (downstream).

In addition to these levels of change, the policy process also must be considered. The framework of Kingdon (8) is useful in illustrating the policy-making process. Kingdon suggests that policies move forward when elements of 3 "streams" come together. (These "streams" are different than the upstream, midstream, and downstream metrics noted above.) The first of these streams is the definition of the problem (eg, a high cancer rate). The second is the development of potential policies to solve that problem (eg, identification of policy measures to achieve an effective cancer control strategy). The third is the role of politics and public opinion (eg, interest groups supporting or opposing the policy). Policy change occurs when a "window of opportunity" opens and the 3 streams push through policy change. A tenet of Kingdon's model is that policy makers are on the receiving end of sometimes disconnected, random, and chaotic data (8,9). Therefore, a key objective of public health practitioners and researchers is to identify metrics for assessing burden, setting priorities, and measuring progress. Such a set of metrics would help public health decision makers as they seek to improve, expand, or terminate policies.

To illustrate the measurement-related issues for public health policy, we review the literature that sets up recommendations. To reach public health goals, we need metrics for the policy environment, just as we do for other environments relevant to public health progress (eg, air, water, the built environment, health care settings).

## Analysis of Metrics in the Literature

### Methods

To better understand the use of policy metrics, we reviewed articles published in 14 public health and preventive medicine journals. The journals chosen were broad, general public health journals and not specific to a single topic such as nutrition or disease. Journals that focused solely on policy and journal supplements were not included. We examined the following journals:

1. *American Journal of Health Behavior*
2. *American Journal of Health Promotion*
3. *American Journal of Preventive Medicine*
4. *American Journal of Public Health*
5. *Australian and New Zealand Journal of Public Health*
6. *Health Education and Behavior*
7. *Health Education Research*
8. *Health Promotion International*
9. *Health Psychology*
10. *Journal of Behavioral Medicine*
11. *Journal of Public Health Management and Practice*
12. *Journal of School Health*
13. *Public Health Reports*
14. *Social Science and Medicine*

We defined a policy article as one that explicitly describes a policy, law, or regulation (including development, implementation, and evaluation). Using online archives, we conducted a systematic audit of articles published in 2008. Tables of contents were collected from each journal issue for that year. Two researchers reviewed the table of contents in each issue and compiled a list of policy-related articles. If the policy content was unclear from the title of the article, the abstract or full text was used. Any articles in question were reconciled by the research team until consensus was reached.

Once the list of policy articles was compiled, the titles were sorted by policy category. To examine policy metrics in detail, 78 articles from 2008 were analyzed. Editorials, commentaries, and reviews were excluded, resulting in 47 articles from which metrics were summarized. For articles that presented data analysis, we assessed policy metrics across several categories:

- the evaluation design
- whether the evaluation was quantitative, qualitative, or both
- the outcome (dependent) variables
- whether metrics were at an upstream, midstream, or downstream level
- whether measurement properties of the metrics were reported
- whether there was specific attention to health disparities
- presence or absence of economic data

### Results

The articles examined were a mixture of both "big P" policy studies (eg, formal laws, rules, regulations enacted by elected officials) and "small p" policy research (eg, organizational guidelines, internal agency decisions or memoranda, social norms guiding behavior) (3). Articles were categorized as child health; maternal health; HIV/AIDS; drug use prevention; tobacco control; violence control; environmental and disaster preparedness and biosecurity; school health; special populations; worksite health; international health; advocacy; general policy; or health care.

The topics that were most represented were tobacco control, international health, and school health. Among international articles, health care was the most common topic. The *Journal of School Health* and the *American Journal of Public Health* published the most policy-related articles.

Most articles (74.5%) relied on a cross-sectional design (Table 1). Only 3 studies reported any economic or cost data. Fourteen studies reported on psychometric properties of the metrics. Most presented new data on psychometric testing (n = 10), while some referred to previous articles (n = 4). The testing most often reported was for reliability (eg, interrater reliability), internal consistency, or key informant validation of methods. When categorizing according to 3 levels of outcomes, most were downstream (n = 31), followed by midstream (n = 13) and upstream (n = 3). Detailed data on health disparities (eg, subgroup analysis for vulnerable populations) were available for only 2 studies. Both of these studies (10,11) explicitly investigated differences among disparate groups; 1 studied how national laws that increased tobacco prices affected smoking prevalence among different socioeconomic groups (by sex, occupation, and birth cohort), and the other investigated differences in the use of skilled birth attendants by women of varying wealth in several countries.

Most of these studies dealt with the effectiveness or evaluation of a given policy that is in effect. Three studies focused on characteristics of or influences on policies that are successfully "passed."

## Recommendations for Policy-Related Metrics

### Expand sources of evidence

Policy outcomes can be monitored by accumulating evidence from many sources to gain insight into a particular topic, often combining quantitative and qualitative data to understand content and track progress. Consensus on valid and useful measures is needed (12). Successfully monitoring outcomes will also require sources beyond the usual public health data sets (eg, tax revenue, polling, and marketing data). We used the 3 domains of evidence-based policy (process, content, outcome) to present sample metrics across the 3 domains (Table 2). Metrics are quantitative (eg, the percentage of the population with a particular health behavior) and qualitative (eg, the content of a certain policy). Most studies in this review were cross-sectional; stronger study designs are needed to improve the evidence base.

### Consider the paradox of local policy evidence

Although much of the effect of public health policy occurs locally, in many jurisdictions high-quality data are lacking at the city, county, or metropolitan levels. Some attempts have been made to identify local-level indicators (13), but a set of consensus policy metrics needs to be developed for local areas, as has been done at the national and state levels.

### Develop systems for policy surveillance

A public health adage is "what gets measured gets done" (14). This has typically been applied to downstream endpoints; however, for policy approaches, midstream and upstream metrics are needed. A few efforts are under way to develop public health policy surveillance systems. For example, a group of federal and voluntary agencies has developed policy surveillance systems for tobacco, alcohol, and more recently, school-based nutrition and physical education (3).

### Increase understanding of practice-based evidence

Policy-relevant evidence should come from settings and organizations that reflect public health practice and policy. For example, efforts such as the Steps to a HealthierUS initiative, YMCA's Activate America, and faith-based interventions demonstrate that existing approaches for leadership development can enhance the use of evidence for promoting physical activity (15). As these efforts are documented, specific attention should be given to the key metrics for measuring progress.

### Make research more accessible for policy audiences

Researchers and policy makers sometimes exist in parallel universes because of decision-making differences, poor timing, ambiguous findings, and lack of relevant data (16). Metrics may become relevant to policy makers when the effects of a health outcome are framed in terms of the direct impact on one's community, family, or constituents (17). An excellent example comes from the Rudd Center Revenue Calculator (www.yaleruddcenter.org/sodatax.aspx), which shows the revenue that could be generated from a 1-cent excise tax per ounce of sugar-sweetened beverages by state or municipality.

### Improve and clarify metrics relevant to health disparities

Eliminating health disparities is a policy imperative. To achieve this goal, we need to better articulate the key domains of inequality. For example, variables have included race/ethnicity, socioeconomic status or social class, geography, age, and sex (18). Our review of the existing literature showed sparse attention to metrics for health disparities and policy.

### Improve incorporation of economic metrics

In deciding whether to take action and how to prioritize resources, policy makers often ask 3 questions: 1) Is there a problem? 2) Do we know how to fix the problem? and 3) How much will it cost? We probably have the most data for answering the first question (19), an intermediate amount for the second (20), and the least data for the economic issues (21). Studies of disease burden that use comparative units of analysis (eg, quality-adjusted life years) provide a basis for economic evaluations (22). Since much of the literature on pay-for-performance has focused on financial incentives, more work is needed to understand how the concepts apply to population-level public health policy.

### Learn by analogy

Although public health research and practice are often segregated into "silos" because of categorical funding streams and interest groups (23), much can be learned across content areas. For example, several authors have examined the lessons from tobacco control that can be applied to the obesity epidemic (24,25). Similar areas in public health where policy measurement is advanced may provide beneficial insights to developing topics.

## Conclusion

Much of what has been learned from surveillance of diseases and risk factors can probably be applied in the policy arena. A full spectrum of outcomes is needed spanning upstream, midstream, and downstream domains. Arriving at these metrics will require creative thinking and application of alternative study designs. For example, adherence to a strict hierarchy of study designs may reinforce an "inverse evidence law" by which interventions most likely to influence whole populations (eg, policy change) are least valued in an evidence matrix emphasizing randomized designs (26). To establish a system that rewards policies for improved population health (5), considerable work is needed on the appropriate metrics.

## Figures and Tables

**Table 1:** Summary of Policy Study Designs and Metrics From Articles in Selected Journals,^a^ 2008^b^

Content Area	No. of Papers	No. With Original Data	No. With Cross-Sectional Design	No. With Outcome Level^c^		

				Upstream	Midstream	Downstream
Child health	2	2	2^d^	1	0	1
Maternal health	0	NA	NA	NA	NA	NA
HIV/AIDS	2	2	2	0	1	1
Drug use prevention	1	1	1	0	1	0
Tobacco control	21	19	14^d^	2	4	15
Violence control	1	1	1	0	0	1
Environmental and disaster preparedness and biosecurity	2	2	2	0	0	2
School health^e^	4	4	3	0	3	1
Special populations	1	1	0	0	1	0
Worksite health	2	1	2	0	2	0
International health	9	7	7^d^	0	1	8
Advocacy	0	NA	NA	NA	NA	NA
General policy	1	1	1	0	0	1
Health care	1	1	0	0	0	1
**Total**	**47**	**42**	**35**	**3**	**13**	**31**

Abbreviation: NA, not applicable.

a *American Journal of Health Behavior*, *American Journal of Health Promotion*, *American Journal of Preventive Medicine*, *American Journal of Public Health*, *Australian and New Zealand Journal of Public Health*, *Health Education and Behavior*, *Health Education Research*, *Health Promotion International*, *Health Psychology*, *Journal of Behavioral Medicine*, *Journal of Public Health Management and Practice*, *Journal of School Health*, *Public Health Reports*, *Social Science and Medicine*.

b Excludes editorials, commentaries, and reviews.

c Upstream interventions involve policy approaches that have the potential to affect large populations through regulation, increasing access, or economic incentives. Midstream interventions occur within organizations, such as worksites. Downstream interventions involve individual-level behavioral approaches for prevention or disease management.

d Includes 1 multilevel study.

e Includes studies on obesity prevention in school settings (eg, wellness policies).

**Table 2:** Metrics for Evidence-Based Public Health Policy Across Various Domains

**Domain**	Objective	Data Sources	Example Metrics for Tobacco Control
Process	To understand approaches to enhance the likelihood of policy adoption	Key informant interviews Case studies Surveys of setting-specific political contexts	Understanding the lessons learned from successful state and local efforts in tobacco control The level of support from policy makers for various tobacco control interventions
Content	To identify specific policy elements that are likely to be effective	Systematic reviews Content analyses	The specific content of model laws on tobacco that make use of decades of research on the impacts of policy on tobacco use The specific content of policies regarding the funding needed for various tobacco control activities (eg, surveillance, health communication, cessation)
Outcome	To document the potential effect of policy	Surveillance systems Natural experiments tracking policy-related endpoints	The changes in rates of self-reported tobacco use The cost-effectiveness of tobacco policy interventions

Source: Adapted from Brownson et al (3).

## References

[1] Institute of Medicine, Committee for the Study of the Future of Public Health (1988). The future of public health.

[2] Centers for Disease Control and Prevention (1999). Ten great public health achievements — United States, 1900-1999. MMWR Morb Mortal Wkly Rep.

[3] Brownson RC, Chriqui JF, Stamatakis KA (2009). Understanding evidence-based public health policy. Am J Public Health.

[4] Christianson JB, Leatherman S, Sutherland K (2008). Lessons from evaluations of purchaser pay-for-performance programs: a review of the evidence. Med Care Res Rev.

[5] Kindig DA (2006). A pay-for-population health performance system. JAMA.

[6] McKinlay JB (1998). Paradigmatic obstacles to improving the health of populations — implications for health policy. Salud Publica Mex.

[7] (2000). State and local legislative action to reduce tobacco use. Smoking and tobacco control monograph no. 11.

[8] Kingdon J (1995). Agendas, alternatives, and public policies.

[9] McDonough J (2000). Experiencing politics. A legislator's stories of government and health care.

[10] Helakorpi S, Martelin T, Torppa J, Vartiainen E, Uutela A, Patja K (2008). Impact of the 1976 Tobacco Control Act in Finland on the proportion of ever daily smokers by socioeconomic status. Prev Med.

[11] Kruk ME, Prescott MR, Galea S (2008). Equity of skilled birth attendant utilization in developing countries: financing and policy determinants. Am J Public Health.

[12] Kindig D, Day P, Fox DM, Gibson M, Knickman J, Lomas J (2003). What new knowledge would help policymakers better balance investments for optimal health outcomes?. Health Serv Res.

[13] Cheadle A, Sterling TD, Schmid TL, Fawcett SB (2000). Promising community-level indicators for evaluating cardiovascular health-promotion programs. Health Educ Res.

[14] Thacker SB (2007). Public health surveillance and the prevention of injuries in sports: what gets measured gets done. J Athl Train.

[15] Cyzman D, Wierenga J, Sielawa J (2009). Pioneering healthier communities, West Michigan: a community response to the food environment. Health Promot Pract.

[16] Brownson RC, Royer C, Ewing R, McBride TD (2006). Researchers and policymakers: travelers in parallel universes. Am J Prev Med.

[17] Jones E, Kreuter M, Pritchett S, Matulionis RM, Hann N (2006). State health policy makers: what's the message and who's listening?. Health Promot Pract.

[18] Kindig DA (2007). Understanding population health terminology. Milbank Q.

[19] Brownson RC, Baker EA, Leet TL, Gillespie KN (2003). Evidence-based public health.

[20] Zaza S, Briss PA, Harris KW (2005). The Guide to Community Preventive Services: what works to promote health?.

[21] Carande-Kulis VG, Maciosek MV, Briss PA, Teutsch SM, Zaza S, Truman BI (2000). Methods for systematic reviews of economic evaluations for the Guide to Community Preventive Services. Task Force on Community Preventive Services. Am J Prev Med.

[22] McAlearney AS, Schweikhart SB, Pathak DS (1999). Quality-adjusted life-years and other health indices: a comparative analysis. Clin Ther.

[23] Wiesner PJ (1993). Four diseases of disarray in public health. Ann Epidemiol.

[24] Green LW, Orleans CT, Ottoson JM, Cameron R, Pierce JP, Bettinghaus EP (2006). Inferring strategies for disseminating physical activity policies, programs, and practices from the successes of tobacco control. Am J Prev Med.

[25] Mercer SL, Green LW, Rosenthal AC, Husten CG, Khan LK, Dietz WH (2003). Possible lessons from the tobacco experience for obesity control. Am J Clin Nutr.

[26] Nutbeam D (2003). How does evidence influence public health policy? Tackling health inequalities in England. Health Promot J Austr.

